# A polymicrobial perspective into the ecological role of *Enterococcus faecalis* in dental root canal infections

**DOI:** 10.1038/s41522-025-00722-w

**Published:** 2025-05-22

**Authors:** Ana Parga, Jade Mattu, Georgios N. Belibasakis, Kimberly A. Kline, Julian G. Leprince, Daniel Manoil

**Affiliations:** 1 Department of Microbiology and Parasitology | Aquatic One Health Research Centre – Faculty of Biology | Universidade de Santiago de Compostela, Santiago de Compostela, Spain; 2 Department of Microbiology and Molecular Medicine | Faculty of Medicine | University of Geneva, Geneva, Switzerland; 3 DMD student | University Clinics of Dental Medicine | Faculty of Medicine | University of Geneva, Geneva, Switzerland; 4 Division of Oral Health and Periodontology | Department of Dental Medicine | Karolinska Institutet | Huddinge, Stockholm, Sweden; 5 Division of cariology and endodontology | University Clinics of Dental Medicine | Faculty of Medicine | University of Geneva, Geneva, Switzerland

**Keywords:** Dentistry, Biofilms, Bacteria, Microbial communities, Microbial ecology, Microbiome

## Abstract

*Enterococcus faecalis*, a non-oral nosocomial pathogen, intriguingly ranks among the most frequently retrieved species from polymicrobial infections of dental root canals. This review integrates findings from the latest omics approaches, alongside emerging evidence of *E. faecalis* interactions within oral polymicrobial communities, to refine our understanding of its role in these infections. Herein, *E. faecalis* emerges as an ecologically invasive species and a catalyst of the pathogenicity of entire communities.

## Introduction

Dental root canal infections are caused by the bacterial colonisation of the dental pulp space and the establishment of polymicrobial biofilms onto the root canal walls^1^. The migration of bacteria and their by-products through apical foramina into periapical tissues causes an inflammation-driven osteolytic lesion, i.e., an *apical periodontitis* (AP)^2^. AP lesions represent a considerable burden, as they are a leading cause of dental emergencies accounting for thousands of hospitalisations annually in the USA^3^, and may exacerbate systemic conditions such as diabetes mellitus II or cardiovascular diseases^4,5^. Since the identification of bacteria as the primary aetiological agents in AP^6–8^, extensive research has attempted to characterise the causal microbial communities^9–11^. Early microbiological investigations, which relied on culture and closed-ended molecular methods, consistently described endodontic communities to represent a subset of around 50 taxa also found in the oral microbiota^12^. As such, endodontic infections appeared to behave as ecological bottlenecks that select among oral taxa those most fit to colonise the root canal environment^1,13^.

These investigations also pointed out one gram-positive bacterium identified in notably high prevalence in roots with failed endodontic treatments; *Enterococcus faecalis*^14–20^. These findings were remarkable primarily because the taxon is not a common member of the oral microbiota but is better known as a leading nosocomial pathogen^21^. Furthermore, its association with post-treatment AP suggested a potential ability to resist standard endodontic procedures, and raised the possibility of a pathogenic role of the taxon in endodontic infections^22^. These findings spurred targeted research aiming to decipher the mechanisms underpinning *E. faecalis* persistence in treated root canals^23–26^. These investigations identified several coping mechanisms likely accounting for its remarkable adaptation to the environment of endodontic infections, somewhat refining the role of the taxon to that of a *survival specialist* rather than an *endodontic pathogen*^23–25,27^. The topic of *E. faecalis* as an individual endodontic taxon and its survival mechanisms have since been comprehensively reviewed^22,28,29^.

More recently, the advent of -omics technologies has revealed a previously unrecognised microbial diversity within infected roots^30,31^, highlighting the contribution of polymicrobial interactions to the pathogenicity of endodontic communities^32^. As these findings underscored the importance of *community-wide pathogenicit*y in endodontic infections, they raised interest in reviewing the role of *E. faecalis* from a broader ecological perspective. Specifically, this review explores the origins of the taxon within the oral microbiome, integrates the latest -omics findings to update the taxon’s ecological place, and discusses evidence on *E. faecalis* interactions within endodontic polymicrobial communities. The evidence reviewed herein may shed a novel light on the ecological role of *E. faecalis* in endodontic infections, balancing the notions of *endodontic pathogen* or *survival specialist*, and is likely to translate more broadly to polymicrobial infections outside of oral niches.

## From the oral microbiome to root canals: on the origins of *E. faecalis*

In microbial ecological terms, root canals with necrotic pulp tissue constitute bleak environments characterised by low oxygen tension and scarce nutrient sources, and even more so after they have been treated, i.e., cleaned from tissue debris and repeatedly exposed to alkaline disinfectants^1^. It is, however, in such an unsupportive environment that *E. faecalis* survives in higher prevalence than other endodontic taxa^14–20^. Several factors likely contribute to its enhanced survival in treated root canals, including: (i) its tolerance to alkaline stress via proton uptake to maintain cytosolic homoeostasis^24,25^, (ii) its survival to long starvation periods by entering a “viable but non-culturable state”^27,33^, (iii) its biofilm formation abilities^34–36^, (iv) its expression of specific adhesins to dentine^34,35^, and (v) its capacity to thrive as single-species without metabolic contingency on other bacteria^22^.

While these traits help explain the increased detection of *E. faecalis* in treated root canals, they overlook the question of the species’ source. Indeed, the selection bottleneck created by the unique microenvironment of the infected root canal can only select from those taxa already present within the oral ecosystem. However, *E. faecalis* is inconsistently found in the oral cavity, and when detected, ranks among the low-abundant taxa. Specifically, cross-sectional culture-based studies report a low prevalence of *E. faecalis* in the oral microbiome ranging from 1 to 17%^37–39^; a carriage that appears to be transient and dependent on the level of oral hygiene^37,40,41^. In terms of abundance, 16S rRNA gene sequencing studies estimate the genus *Enterococcus* spp. to represent only around 1.3% of all 16S sequences catalogued in the Human Oral Microbiome Database (HOMD), a reference repository for oral microbial taxonomy^42,43^. The scarcity of *E. faecalis* in the oral ecosystem contrasts with its much higher recovery rate from infected root canals, which ranges from 24 to 90% as determined by culture or polymerase chain reaction (PCR) techniques^14,19,44,45^. To explore this discrepancy, several studies have attempted to identify genetic relationships between *E. faecalis* strains isolated from saliva and their endodontic counterparts^39,46,47^. While some data described genetic differences between salivary and endodontic isolates^46^, other evidence identified similarities, pointing to saliva as a potential contaminating source^47^. A recent study using whole-genome sequencing (Illumina and Nanopore technologies) found that *E. faecalis* strains from saliva and root canals isolated from the same patients clustered phylogenetically, further supporting the idea that saliva serves as a reservoir for *E. faecalis*^39^. In this latter study, the presence of *E. faecalis* in saliva was also associated with its increased detection in root canals, and higher odds of having post-treatment AP^39^. But if indeed endodontic *E. faecalis* isolates originate from saliva, how then to reconcile the low prevalence and abundance of the taxon in the oral microbiome with its high detection rates in root canals? A plausible explanation lies in an exogenous source of *E. faecalis* combined with a transient colonisation of the oral ecosystem.

Outside its natural niches in the gastrointestinal tracts of mammals and birds, *E. faecalis* is commonly found in fermented and dairy products^48^. Its presence in these foods is often-times intentional, as it is employed as a starter culture in fermentation processes^49^, although it may also appear as a contaminant in processed meats owing to the typical sturdiness and high-temperature resistance of the species^50,51^. A foodborne colonisation could explain the sporadic presence of *E. faecalis* in the oral cavity, assuming the taxon can transiently overcome the resilience of the oral microbiota, i.e., bypass the ability of oral communities to maintain, or regain, their taxonomic and metabolic profiles^52^. There is evidence to support this hypothesis. *E. faecalis* was shown to be able to integrate into a six-species oral biofilm model, where it could grow in high abundance and hinder the growth of typical oral commensals such as *Actinomyces oris* and *Streptococcus mutans*^53^. This behaviour appears to translate to in vivo conditions. To explore *E. faecalis*’ foodborne origin, Swiss researchers assessed the load of the taxon in various commercially available cheeses, and monitored over time the colonies retrieved from oral rinses of participants who ingested a cheese portion containing approx. 5 × 10^5^ *E. faecalis* colony forming units (CFU)^40^. While no *E. faecalis* were retrieved from participants prior to cheese ingestion, median CFUs of 5 × 10^3^, 1 × 10^3^, and 1 × 10^2^ were still detected after 1, 10, and 100 min, respectively. CFUs dropped below detection limits within one week. These findings support the ability of *E. faecalis* to transiently persist within the oral cavity. In fact, this methodology may even have somewhat underestimated *E. faecalis*’ persistence, as only planktonic cells were retrieved from mouth rinses, thereby adding a dilution factor and overlooking cells that potentially adhered to tissues and other bacteria.

To account for such adhered cells, another study specifically addressed the recovery of foodborne *E. faecalis* from oral biofilms after consumption of enterococci-containing cheeses^54^. To do so, dental splints with enamel slabs were placed in the oral cavities of six volunteers either three days prior to, or on the day of, cheese consumption. Slabs placed three days in advance were utilised to assess the integration of *E. faecalis* into naturally formed oral biofilms as compared to its adhesion onto pristine slabs. Five days after cheese ingestion, all slabs were removed, and the presence of *E. faecalis* within the formed biofilms was identified by culture and by fluorescent in situ hybridisation (FISH). *E. faecalis* cells were observed and recovered from the biofilms of all participants but one, in similar numbers whether they attached onto pristine enamel slabs or pre-colonised ones. These findings support a potential colonisation of the oral ecosystem by foodborne *E. faecalis*, and specifically underscore a potential anchoring role of oral biofilms.

Taken together, current evidence supports the foodborne colonisation as a plausible source of *E. faecalis* in the oral microbiome. *E. faecalis’* establishment appears to be in competition with oral commensals, yet able to transiently overcome the resilience of oral communities. This transient colonisation may result in a “cycling” pattern, where *E. faecalis* cells present at a given time point could infect and persist within root canals, while those remaining in the oral cavity would undergo a turnover and be gradually replaced. Sucha transient colonisation pattern would reconcile studies that identified distinct *E. faecalis* genotypes in saliva and root canals. Verifying this hypothesis would, however, require longitudinal studies able to monitor “cycles” of transient *E. faecalis* colonisation, and match oral isolates to those from concurrent endodontic infections.

## *E. faecalis* in endodontic infections through the prism of omics

Prior to the advent of next-generation sequencing (NGS) approaches (understand high-throughput DNA sequencing of either short or long reads), the microbial composition of endodontic infections was primarily explored using culture and close-ended molecular methods, including PCR, DNA-DNA chequerboards or FISH^10,14,44,55^. These classical approaches typically identifying sets of 30 to 50 species in primary, and 20 to 30 species in post-treatment endodontic infections^56,57^. Primary infections were typically dominated by strictly anaerobic, proteolytic species, such as *Fusobacterium nucleatum*, *Porphyromonas* spp., *Prevotella* spp., *Treponema* spp., and several Bacillota, including *Pseudoramibacter alactolyticus*, *Dialister pneumosintes*, and *Parvimonas micra*. In contrast, secondary infections exhibited higher prevalences of facultatively anaerobic, saccharolytic species, notably a diverse range of *Streptococcus* spp. and *Actinomyces* spp^12^. In the majority of these studies, *E. faecalis* was outlined as a species occasionally found in primary infections, but that could reach prevalences ranging between 24 and 90% in post-treatment cases^14,18,19,45,58^.

Neither limited by the challenges of culturing, nor predetermined by the selection of primers and probes, NGS outcomes unravelled an unanticipated bacterial diversity. Overall, more than 500 distinct species-level taxa were identified across studies, mostly inferred from 16S amplicon sequencing pipelines applying a 97% similarity threshold for taxonomic assignment^30^. This overall charting of root canals’ diversity is to be distinguished from the ecology of individual infections, shown to harbour between 8 and 460 distinct species^59,60^. This comprehensive taxonomic mapping provided several key insights. Most notably, it enabled the assessment of relative abundance, i.e., the quantitation of each taxon relative to the entire community, a measure largely inaccessible to classical methodologies. Within this expanded ecological framework, distinct clinical presentations of endodontic infections were linked to differentially abundant microbial communities, where no taxon was unequivocally specific of distinct infection types^30,61^.

These technological advances complemented and extended previous observations on the ecological place of *E. faecalis*. NGS data confirmed the occasional detection of *E. faecalis* in primary infections, which always ranked among low-abundant taxa (<1%) (Table 1)^62–71^. In post-treatment infections, NGS detected the taxon in prevalences rarely exceeding 50% (Table 1), somewhat moderating the high values previously reported by classical methodologies, nevertheless confirming a higher prevalence of the taxon than in primary infections^59,60,64,66,68–70,72–75^. When present, however, the taxon averaged relative abundance between 5 and 20%^59,66,70,72–74^. It is worth emphasising that even the lower end of this range represents significant dominance within a highly diverse microbial community typically comprising over 500 species-level taxa. For perspective, if every species occupied 5% of reads, one could only “fit” 20 species within an infected root canal, underscoring the ability of *E. faecalis* to outcompete other taxa under challenging conditions. Beyond averages, the taxon’s relative abundance spiked over 25% in certain individual samples^59,60,70,73,74^. This identification pattern, i.e., inconsistently detected yet dominant when present (Table 1), was effectively exemplified in one hierarchical cluster analysis of taxa found in post-treatment infections^59^. In this analysis, while only 11/22 samples displayed *E. faecalis*, its relative abundance sporadically exceeded 99% in some specimens–evocative of early culture-based studies identifying *E. faecalis* as a mono-infectant^76^. This paroxysmal pattern aligns and supports the hypothesis of a foodborne and transient colonisation of the oral ecosystem mentioned above.

**Table 1:** *E. faecalis* representation in metataxonomic and metagenomic studies of endodontic infections

Study	Technology	Endodontic diagnosis	Number of samples	*E. faecalis* prevalence	Mean *E. faecalis* abundance
Santos et al.^121^	16S sequencing^1^ Pyrosequencing	PEI	8	NR	NR
Siqueira et al.^122^	16S sequencing^1^ Pyrosequencing	PEI	10	NR	NR
Hsiao et al.^62^	16S sequencing^1^ Pyrosequencing	PEI	16	6.25%^4^	NR
Özok et al.^63^	16S sequencing^1^ Pyrosequencing	PEI	23	78%^4^	0.2%^4^
Anderson et al.^60^	16S sequencing^1^ Pyrosequencing	PTEI	40	17.5%	2.6%
Hong et al.^123^	16S sequencing^1^ Pyrosequencing	PEI PTEI	10 8	NR NR	NR 0.7%^4^
Vengerfeldt et al.^64^	16S sequencing^1^ Illumina HiSeq 2000	PEI PTEI	5 3	0% 33%	0% NR
Gomes et al.^65^	16S sequencing^1^ Illumina MiSeq	PEI	15	22%^4^	NR
Tzanetakis et al.^124^	16S sequencing^1^ Pyrosequencing	PEI PTEI	24 24	NR NR	0.8%^4^ 1.3%^4^
Siqueira et al.^72^	16S sequencing^1^ Illumina MiSeq	PTEI	10	40%	<5%
Keskin et al.^66^	16S sequencing^1^ Pyrosequencing	PEI PTEI	40 20	75% 80%	2%^4^ 5%^4^
Persoon *et al*^93^	16S sequencing^1^ Illumina MiSeq	PEI	23	NR	NR
Bouillaguet et al.^59^	16S sequencing^1^ Illumina MiSeq	PEI PTEI	21 22	NR >50%	<0.04% 18.9%
Sánchez-Sanhueza et al^125^	16S sequencing^1^ Illumina MiSeq	PTEI	24	NR	NR
Zandi et al.^73^	16S sequencing^1^ Pyrosequencing	PTEI	10	20%	13.9%
Manoharan et al.^67^	16S sequencing^1^ Illumina MiSeq	PEI	32	<20%	NR
Amaral et al.^126^	16S sequencing^1^ Illumina MiSeq	PEI	25	NR	NR
Zhang et al.^127^	16S sequencing^1^ Illumina NovaSeq	PTEI	10	NR	NR
Buonavoglia et al.^68^	16S sequencing^2^ Nanopore MinION	PEI PTEI	8 9	12.5% 11.1%	NR NR
Ordinola-Zapata et al.^69^	16S sequencing^1^ Illumina MiSeq	PEI PTEI	31 27	6.5% 3.7%	<0.001% 0.6%
Pérez-Carrasco et al.^74^	16S sequencing^1^ Illumina MiSeq	PTEI	21	38%^4^	4.8%^4^
Abraham et al.^70^	16S sequencing^1^ Ion Torrent	PEI PTEI	10 10	20% 70%	NR <6%
Alquria et al.^71^	16S sequencing^1^ Illumina MiSeq	PEI	27	<25%^4^	<10%^4^
Arias-Moliz et al.^128^	16S sequencing^1^ Illumina MiSeq	PTEI	32	NR	NR
Ordinola-Zapata et al.^129^	Shotgun sequencing^3^ Illumina NovaSeq	PEI PTEI	22 18	NR NR	NR NR
Park et al.^75^	16S sequencing^1^ Illumina MiSeq	PEI PTEI	10 10	NR 30%	0.02% 1.15%

*NR* not reported. The table compiles prevalence and abundance values of *E. faecalis* as identified by NGS technologies. Data extraction considered intra-radicular samples of either primary endodontic infections (PEI) or post-treatment endodontic infections (PTEI). Whereas some studies also included outcomes from various periapical lesions, including abscesses or granulomas, these were not extracted herein to enhance homogeneity and allow potential comparisons between studies.

^1^ Partial length sequencing of the 16S rRNA gene (targeted variable regions).

^2^ Full length sequencing of the 16S rRNA gene.

^3^ Shotgun metagenomic sequencing (read-based taxonomy mapping).

^4^ Values reported refer to the genus *Enterococcus* spp.

Studies in this table are drawn from a previously published systematic review by our group, and complemented manually to incorporate the latest research using the same search methodology^30^. Few studies were excluded from this table based on the previous risk of bias assessment. These include Li et al.^130^, Iriboz et al.^131^ and Qian et al.^132^. Additionally, Kumari et al.^133^ was excluded because the study failed to comply with the Declaration of Helsinki on Ethical Principles for Medical Research Involving Human Subjects – Articles 11 and 23 (breach of confidentiality)^134^.

Overall, insights into the relative abundance of *E. faecalis* represent one of the most significant contributions of NGS to our understanding of the taxon’s ecology within endodontic communities, suggesting an invasive behaviour when present. Several considerations are, however, worth mentioning when discussing *E. faecalis*’ abundance. While roughly half of the studies reviewed herein identified the taxon to the species level, the rest only resolved taxonomy down to the genus level, hence potentially overestimating *E. faecalis*-specific reads, as other enterococcal species such as *E. faecium, E. casseliflavus* and *E. durans*, may also contribute to the community composition–though less prevalent (Table 1)^77,78^. Also, while relative abundance is a rather accurate proxy to estimate ecological fitness, it hardly informs on a taxon metabolic function within the community, which would rather be addressed by means of meta-transcriptomics or -proteomics.

Thus far, few studies applied such functional approaches to endodontic infections (Table 2). One metatranscriptomic study that investigated both primary and post-treatment infections, as well as one metaproteomic study that focused on primary infections did not report any transcripts or peptides of enterococcal origin altogether^79,80^. Two other studies characterising the metaproteome of post-treatment infections did identify enterococcal proteins^81,82^, sometimes even representing over 50% of all peptides^81^. Although no peptides mapped against known virulence factors in these two studies, the predominance of enterococcal peptides underscored an important metabolic activity of the taxon within these endodontic communities. These findings fairly align with another study that analysed the metaproteome of both primary and post-treatment infections indiscriminately^83^. Their outcomes confirmed the presence of enterococcal peptides in 40% of the samples, and highlighted several virulence-associated peptides, together with peptides involved in antibiotic resistance and *horizontal gene transfer* (HGT) (Table 2).

**Table 2:** *E. faecalis* representation in metatranscriptomic and metaproteomic studies of endodontic infections

Study	Technology	Endodontic diagnosis	Number of samples	Findings
Nandakumar et al.^83^	LC-MS/MS	PEI & PTEI	4 (PEI) 3 (PTEI) Analysed indiscriminately	Of 89 total proteins resolved, 57 were of enterococcal origin. *E. faecalis*-specific proteins were identified in 43% of samples. Among which: virulence factors (aggregation substance PrgB, hemolysin A, extracellular serine protease), ABC transporters (EmrB/QacA, Lantibiotic permease), antibiotic resistance (PBP4, metallo β-lactamase, vancomycin sensors, TetM, TetT, pheromone PrgE, conjugal transfer proteins).
Provenzano et al.^80^	nanoLC-MS/MS	PEI	6	No *E. faecalis-*specific proteins were identified among all bacterial proteins.
Provenzano et al.^81^	nanoLC-MS/MS	PTEI	10	Enterococcal proteins were identified in 90% of samples, proteins of this genus were the most abundant overall.
Francisco et al^82^	LC-ESI-MS/MS	PTEI	20	23 proteins of *Enterococcus* spp. were identified.
Pinheiro et al^79^	cDNA shotgun sequencing Illumina NovaSeq	PEI	5	No *E. faecalis* transcripts were identified among all bacterial transcripts.
		PTEI	5	No *E. faecalis* transcripts were identified among all bacterial transcripts.

*LC-MS/MS* Liquid Chromatography-tandem Mass Spectrometry.

*ESI* Electrospray Ionisation.

Studies in this table are drawn from a previously published review by our group^31^. A complementary search has additionally identified Pinheiro et al. ^79^.

The table highlights main findings on *E. faecalis* as identified by meta-transcriptomic and -proteomic approaches. Data extraction considered intra-radicular samples of either primary endodontic infections (PEI) or post-treatment endodontic infections (PTEI). Whereas some studies also included outcomes from various periapical lesions, including abscesses or granulomas, these were not extracted herein to enhance homogeneity and allow potential comparisons between studies.

Collectively, these metaproteomic findings complemented NGS data. They confirmed that *E. faecalis* cells may express pathogenic determinants during the course of the infection. Specifically, the identification of multiple antibiotic resistance factors, along peptides involved in HGT, emphasises the rising trend of multi-resistances among enterococci and their potential to disseminate resistance genes.

## Community-wide pathogenicity in endodontic infections: the *E. faecalis* paradigm

While NGS approaches proved instrumental in characterising the full taxonomic diversity of endodontic infections, there is more to the concept of *polymicrobiality* than the blunt enumeration of bacterial taxa. The main relevance of better understanding polymicrobial communities lies in that their pathogenicity cannot be predicted from single taxa. Rather, the pathogenicity of polymicrobial communities depends on their taxonomic composition as specific inter-species interactions modulate the expression of virulence factors that otherwise remain silent in mono-species cultures^32^.

**Fig. 1:**
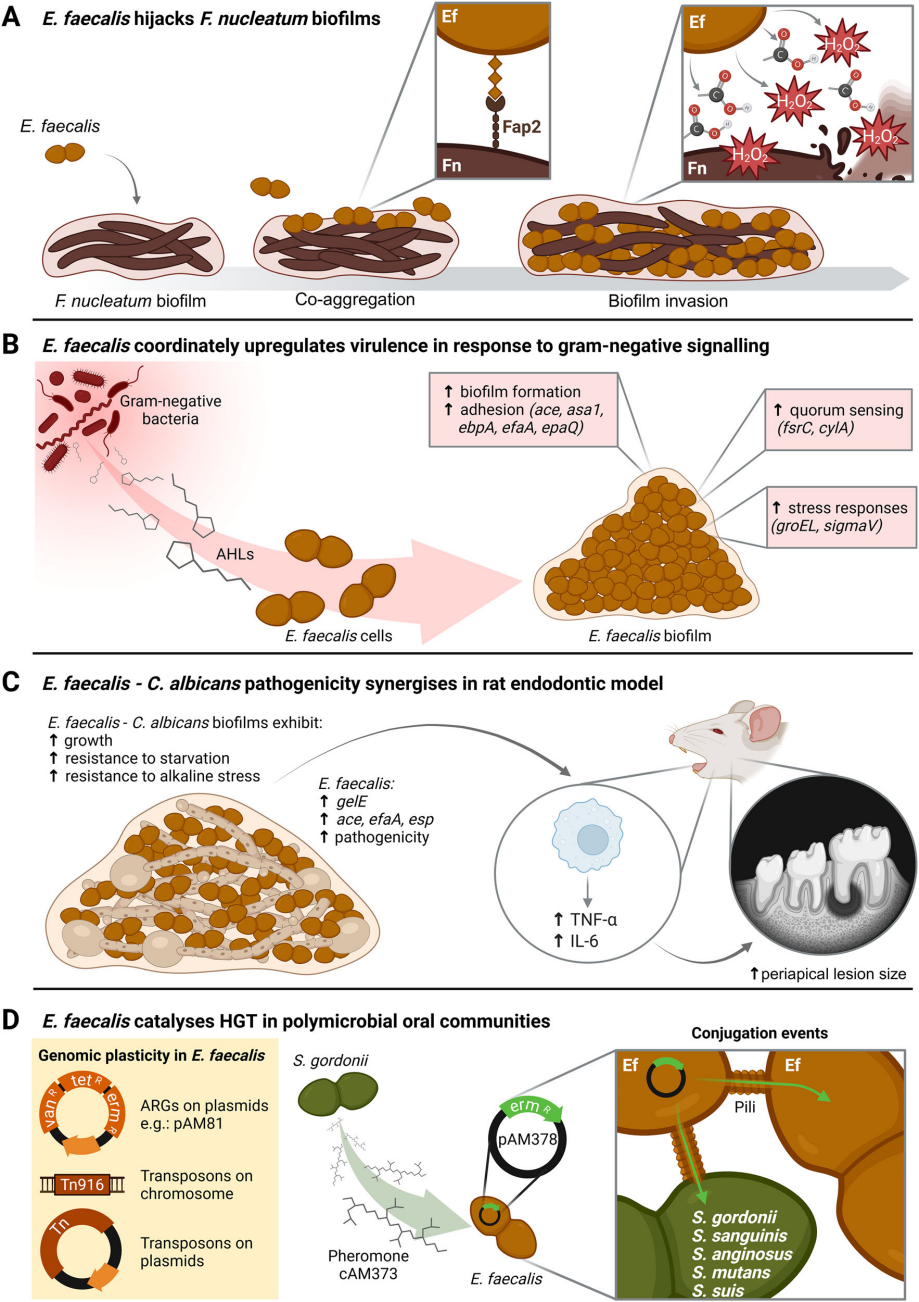
*E. faecalis* pathogenicity is enhanced within polymicrobial communities. Schemes illustrate key synergistic and antagonistic interactions driven by *E. faecalis* within oral and endodontic microbial communities. **A** *E. faecalis* co-aggregates with *F. nucleatum* via the adhesin Fap2. Further metabolic production of organic acids (represented by the carboxylic groups) and hydrogen peroxide (H_2_O_2_) enables ecological competition and deeper biofilm penetration. **B** *E. faecalis* senses AHLs produced by gram-negative bacteria and upregulates a network of genes associated with virulence and host invasion. **C** Dual-species biofilms of *E. faecalis* and *C. albicans* demonstrate enhanced growth and improved resistance to starvation and alkaline stress. Also, endodontic infections combining these two species induce heightened inflammatory responses, resulting in exacerbated periapical lesions in a rat model. **D** *E. faecalis* genome exhibits number of exogenously acquired elements, including multiple conjugative transposons and plasmids that often carry resistance determinants to antibiotics such as, vancomycin, tetracycline, or erythromycin (respectively *van*^*R*^*, tet*^*R*^ and *erm*^*R*^ on the left panel). Additionally, the taxon can respond to peptidic pheromones released by *S. gordonii* and initiate HGT not only between *E. faecalis* cells, but also with several non-pheromone producing oral streptococci (middle and right panels). This figure compiles evidence from multiple scientific references^84,85,87–89,97,100–105^. The web interface BioRender was utilised in the design of this illustration. *AHL* acyl-homoserine lactones, *ARG* antibiotic resistance gene, *HGT* horizontal gene transfer.

The recognised abilities of *E. faecalis* to cope with harsh conditions largely arise from its capacity to establish synergistic or antagonistic interactions with other taxa and to modulate its microenvironment^36^. Whereas *E. faecalis* is known to thrive in distinct niches, spanning from digestive tracts to contaminated surfaces, the taxon can also specifically co-aggregate with oral taxa, and use these anchoring points to further colonise the oral ecosystem. Evidence shows that a majority of oral *E. faecalis* isolates can co-aggregate in vitro with the common endodontic pathogen *F. nucleatum*, and that co-aggregation is specifically mediated by *F. nucleatum*’s Fap2 adhesin^84,85^. In this interaction, *E. faecalis* hijacks *F. nucleatum* biofilms by adhering to Fap2, and then inactivates *F. nucleatum* cells by its inherent metabolic production of organic acids and hydrogen peroxide. This, in turn, promotes the deeper penetration of *E. faecalis* cells into the biofilm, ultimately replacing *F. nucleatum* (Fig. 1A)^85^. Interestingly, such ecological antagonism translates in vivo, as observed in co-occurrence analyses that demonstrate negative correlations between *E. faecalis* and *F. nucleatum* in the ecosystem of endodontic infections^59^.

Beyond mere ecological competition, such “biofilm invading” phenotype can also result from the coordinated transcription of specific virulence traits. This was demonstrated in vitro using a biofilm model co-culturing distinct oral *E. faecalis* strains along three endodontic isolates of the species *Actinomyces naeslundii*, *Lactobacillus salivarius* and *Streptococcus gordonii*^86^. In this model, an *E. faecalis* strain that expressed elevated levels of gelatinase (GelE) and serine protease (SprE) suppressed the proliferation of *L. salivarius* and *S. gordonii* resulting in biofilms dominated by *E. faecalis* and *A. naeslundii*. Evidence further indicates that *E. faecalis* can differentially express virulence traits in response to cues from other bacteria without need of direct contact. A recent study showed, indeed, that *E. faecalis* is able react to acyl-homoserine lactones; signalling molecules long considered the monopole of gram-negative cell-cell communication^87^. In several *E. faecalis* strains, exposure to these gram-negative cues enhanced biofilm formation and upregulated a network of genes able to heighten virulence and host invasion properties. This network included several adhesins (*ace, asa1, ebpA, efaA*), the glycosyltransferase (*epaQ*), stress response proteins (*sigmaV, groEL*), a two-component system (*fsrC*) and a cytolysin (*cylA*) (Fig. 1B).

In other instances, it is synergistic interactions, rather than competitive behaviours, that promote *E. faecalis* virulence within polymicrobial communities. A study showed that *E. faecalis* better resists nutrient deprivation and starvation when co-cultured in two-species biofilms containing either *Candida albicans, S. gordonii, Actinomyces viscosus*, or *Lactobacillus acidophilus*^88^. This effect was especially marked with *C. albicans*. This synergism was highlighted in another study that co-cultured *E. faecalis* and *C. albicans*^89^. In this model, dual-species in vitro biofilms demonstrated mutually enhanced growth and improved survival to high pH stresses, while *E. faecalis* showed upregulation of several virulence-associated adhesins (*ace, efaA, esp*) and a protease (*gelE*). Furthermore, co-infections with these taxa in a rat endodontic infection model were shown to induce increased IL-6 and TNF-α responses and cause significantly more extended periapical lesions (Fig. 1C)^89^. These observations hold clinical significance as *C. albicans* is a fairly common find in post-treatment endodontic infections, with prevalence ranging from 0.5 to 55%, where it is known to co-occur with acidogenic bacteria such as *E. faecalis*^90–93^.

Another pathogenic aspect arising from the close-knit interactions within oral and endodontic polymicrobial communities is their propensity to exchange genetic material^94^. HGT plays a critical role in this process, as many mobile genetic elements, such as plasmids and transposons, often carry virulence-associated and antibiotic resistance genes (ARGs)^95,96^. This holds particular relevance for *E. faecalis*, whose remarkable genomic plasticity allows the taxon to readily acquire and transfer such mobile genetic elements, which can make up to over a quarter of the genome of some strains^97–99^. These abilities were exemplified in an in vitro endodontic model used to assess the conjugation rates between *E. faecalis* and *S. gordonii* of pAM81; a plasmid conferring erythromycin resistance^100^. Authors transformed the plasmid into naïve cells of either *E. faecalis* or *S. gordonii* and co-cultured them in dental roots, alternating resistant mutants with naïve representatives of each species. Under erythromycin selection, taxon identification from the infected roots revealed a bi-directional exchange of the plasmid between the two species. Such conjugative transfers are bolstered in *E. faecalis* by co-resident oral streptococci. A study demonstrated that *S. gordonii* releases a pheromone capable of inducing a conjugative response in *E. faecalis* cells carrying the plasmid pAM378, which frequently harbours ARGs to vancomycin and tetracycline^101^. Remarkably, exposure of pAM378-*E. faecalis* cells to this pheromone triggered transconjugation events not only to *S. gordonii*, but also to non-pheromone-producing streptococci, such as *S. mutans*, *Streptococcus sanguinis*, *Streptococcus anginosus*, and *Streptococcus suis* (Fig. 1D).

These observations parallel the well-documented mobilisation of *E. faecalis’* transposon Tn916, which is now widespread amid oral communities^102^. Tn916 carries a variety of resistance cassettes, including ARGs to macrolides, tetracyclines as well as to kanamycin and erythromycin^103–105^. The dissemination of such determinants to a variety of oral commensals may carry severe clinical implications, as many of such recipients include viridans group streptococci that notoriously cause opportunistic infections on heart valves^106^. More critically, the colocation of multiple ARGs on a single mobile element enables their coselection even in the absence of the antibiotics’ exposure, hence contributing to the persistence and spread of antibiotic resistances^107,108^. Taken together, these findings highlight the central role of *E. faecalis* in the dissemination of resistance determinants across taxa^109^. This role of ARG trafficker may hold particular relevance for *E. faecalis* oral isolates, as the oral microbiome may act as a putative exchange platform between known ARG reservoirs including the environment^110^ and the gut microbiota^111^.

Besides mechanistic aspects, antimicrobial resistance surveys underscore the clinical reality of ARG dissemination among endodontic *E. faecalis*^112^. Cumulative evidence from antimicrobial susceptibility testing frequently reveal resistances to several antibiotic families, including tetracyclines, quinolones and chloramphenicol^113–116^. Specifically, tetracycline resistance was reported in 14 to 70% of *E. faecalis* endodontic isolates^113–115^, ciprofloxacin resistance in 15 to 19%^113,116^, and chloramphenicol in approximately 5% of isolates^113,116^. Furthermore, a recent survey of 37 *E. faecalis* isolates identified two clones resistant to both vancomycin and tigecycline^117^. These findings highlight the propensity of clinical clones to acquire multi-resistances, which is especially concerning in the case of tigecycline; a last-resort glycylcycline with resistance prevalence remaining below 0.4% in *E. faecalis* thus far^118–120^. While endodontic infections are primarily managed through the chemo-mechanical debridement of the pulpal space, the identification of resistant strains is all the more troubling as antibiotics in dentistry are normally reserved for infections that spread in adjacent tissues, where a rapid infection control is warranted. It is finally worth noting, however, that the clinical applicability of resistance surveys in endodontics could be enhanced by avoiding tests on antibiotics to which *E. faecalis* is inherently resistant, such as macrolides, lincosamides, or nitroimidazoles, and streamlining research efforts on epidemiologically relevant antibiotics as outlined by international standards such as EUCAST or CLSI.

Altogether, *E. faecalis* emerges as a key player within the complex polymicrobial interplay of oral and endodontic biofilms. The taxon displays a remarkable plasticity in sensing the presence of both competing and cooperative bacteria. In response, *E. faecalis* can alternatively modulate its transcriptional profiles towards increased virulence or become conducive to HGT. Because *E. faecalis* is a known carrier of mobile genetic elements, its ability to easily transfer plasmids and transposons makes of the taxon a potential *catalyst* for the dissemination of virulence traits and ARGs within polymicrobial communities.

## Conclusions

*Survival specialist* or *endodontic pathogen*? The current body of evidence depicts a multifaceted role of *E. faecalis* in endodontic infections. Its acknowledged abilities to withstand harsh conditions endow the taxon with a competitive edge over other taxa that likely account for its ecological selection and dominance, in certain cases of post-treatment infection. Whereas these observations highlight the aptitude of the taxon to survive under adverse conditions, there is also evidence pointing to a more pathogenic role. Typically, despite being allochthonous in the oral ecosystem, *E. faecalis* can adventitiously overcome the resilience of the oral microbiome and colonise it, albeit transiently. Furthermore, the taxon thrives within endodontic polymicrobial communities, some times hijacking surface motifs of oral taxa to overtake the ecological niche, other times leveraging synergistic interactions to enhance its own survival and the pathogenicity of the community.

Yet, because its mere presence in the oral microbiome does not equate to infection, one can make the case for a more nuanced role than that of a mere pathogen. Specifically, its role as a vector in HGT underscores its propensity to disseminate ARGs amid oral communities and broadly impact the community’s virulence. Altogether, *E. faecalis* emerges as an ecologically invasive species and a catalyser of community-wide pathogenicity. Not unexpectedly, the evidence reviewed herein also highlighted several pending questions: What mechanisms drive *E. faecalis* transiency in the oral microbiome? To what extent does it mobilise ARGs across oral taxa? And how easily can these “oral” ARGs disseminate systemically? Addressing these questions may help identify connections between oral *E. faecalis* isolates and clinical clusters commonly recovered from endocarditis or urinary infections, and thereby determine to what extent oral isolates may disseminate to cause more severe infections.

## Data Availability

No datasets were generated or analysed during the current study.
